# Crystal structure of ethyl 2-[2-(4-methyl­benzo­yl)-5-*p*-tolyl-1*H*-imidazol-1-yl]acetate

**DOI:** 10.1107/S2056989016002504

**Published:** 2016-02-17

**Authors:** E. Arockia Jeya Yasmi Prabha, S. Suresh Kumar, Anil K. Padala, Qazi Naveed Ahmed, S. Athimoolam

**Affiliations:** a Department of Physics, University College of Engineering Nagercoil, Anna University, Nagercoil 629 004, India; b Medicinal Chemistry Division, Indian Institute of Integrative Medicine (IIIM) and Academy of Scientific and Innovative Research (AcSIR-IIIM), Jammu 180 001, India

**Keywords:** crystal structure, imidazole derivative, new synthesis, 2-(4-meth­oxy­phen­yl)-2-oxoacetaldehyde, glycine methyl ester hydro­chloride

## Abstract

In the title compound, C_22_H_22_N_2_O_3_, the plane of the five-membered ring is oriented at dihedral angles of 45.4 (1) and 52.5 (1)° to the phenyl rings. Furthermore, this ring makes an angle of 85.2 (2)° with the plane of the ethyl acetate substituent. The mol­ecular structure is affected by an intra­molecular C—H⋯O hydrogen bond between an H atom from the *p*-tolyl group and the carbonyl O atom of the acetate. The methyl group of the ethyl acetate residue is disordered over two sites with equal occupancies. The crystal structure features inter­molecular C—H⋯O and C—H⋯N inter­actions. One of the C—H⋯O hydrogen bonds forms a *C*(5) chain motif extending along the *a* axis. In addition, C—H⋯N contacts form inversion dimers with *R*_2_^2^(12) ring motifs, linking the imidazole ring system to the benzene ring of the *p*-tolyl substituent.

## Abstract 2

The crystal structure of ethyl 2-[2-(4-methyl­benzo­yl)-5-*p*-tolyl-1*H*-imidazol-1-yl]acetate is stabilized by inter­molecular C—H⋯N and C—H⋯O inter­actions.

## Chemical context   

Imidazole and its derivatives have numerous pharmaceutical applications including uses as anti­fungal (Shingalapur *et al.* 2009 ▸), anti­microbial (Sharma *et al.* 2009 ▸), anti-inflammatory (Puratchikody *et al.* 2007 ▸), analgesic (Achar *et al.* 2010 ▸), anti­tubercular (Pandey *et al.* 2009 ▸), anti­depressant (Hadizadeh *et al.* 2008 ▸), anti­leishmanial (Bhandari *et al.* 2009 ▸) and anti­cancer agents (Ozkay *et al.* 2010 ▸). We are inter­ested in the synthesis of active pharmaceutical ingredients (APIs) based on imidazoles and we report here the synthesis and crystal structure of the title imidazole derivative.
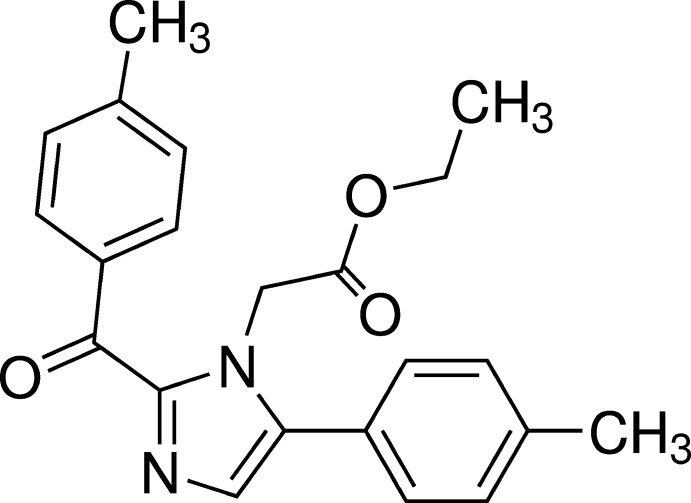


## Structural commentary   

**Figure 1:**
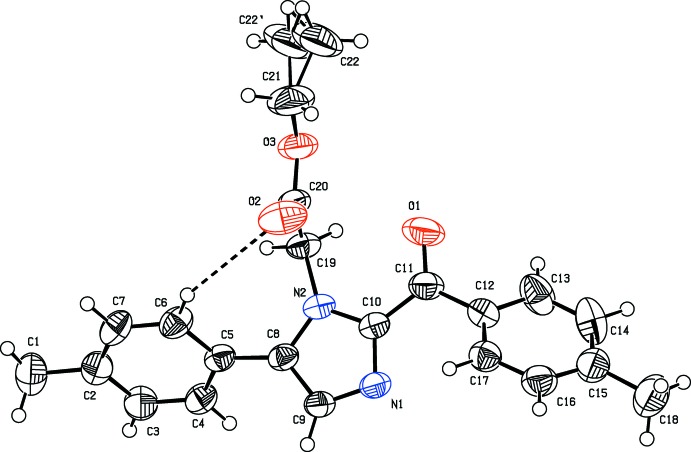
The mol­ecular structure of the title compound, showing the atom-numbering scheme and 50% probability displacement ellipsoids. The methyl group (C22) of the side chain is disordered over two positions each with 0.5 occupancy.

The mol­ecular structure of the title compound is shown in Fig. 1 ▸. The C—N bond lengths within the imidazole ring are 1.373 (3) Å (C10—N2), 1.372 (3) Å (C8—N2), 1.349 (3) Å (C9—N1) and 1.329 (3) Å (C10—N1). These bond distances are shorter than the single-bond length (1.443 Å) and longer than the accepted double-bond length (1.269 Å) due to electron delocalization in the central imidazole ring. The phenyl rings and the plane of the imidazole ring are inclined at angles of 45.4 (1)° (with the C12–C17 ring) and 52.5 (1)° (with the C2–C7 ring). The phenyl rings are oriented to each other with a dihedral angle of 88.1 (1)°. Further, the imidazole ring is inclined at an angle of 85.2 (2)° to the best-fit plane through atoms C19, C20, O3, C21 and C22 of the ethyl acetate substituent. The mol­ecular structure is also influenced by the formation of an intra­molecular C6—H6⋯O2 hydrogen bond, Table 1 ▸, which generates an *S*(8) ring motif (Bernstein *et al.*, 1995 ▸).

## Supra­molecular features   

**Figure 2:**
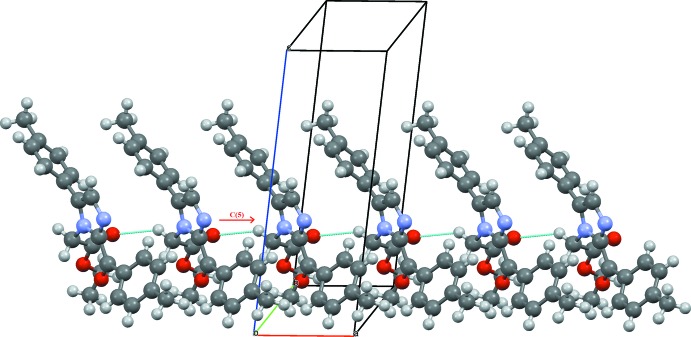
Linear *C*(5) chains formed by a C—H⋯O inter­molecular inter­action extending along the *a* axis of the unit cell.

**Figure 3:**
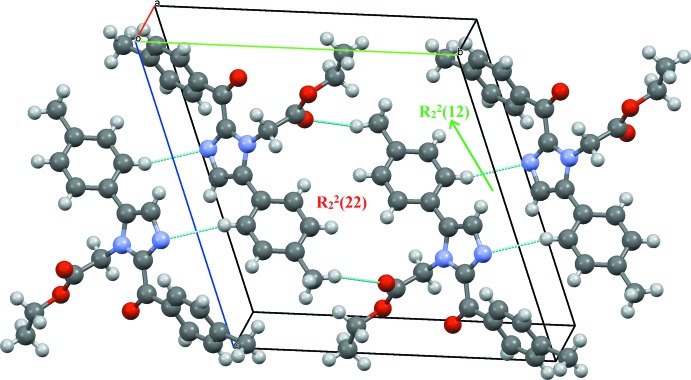
Inversion dimers with 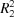
(12) and 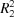
(22) ring motifs resulting from C—H⋯N and C—H⋯O hydrogen bonds.

The N-bound methyl­ene group of the side chain is connected with the carbonyl oxygen of an adjacent mol­ecule through a C19—H19*A*⋯O2 hydrogen bond, forming a linear *C*(5) chain motif along the *a* axis, Table 1 ▸ and Fig. 2 ▸. The phenyl and imidazole rings are linked through inversion-dimer formation involving C4—H4⋯N1 hydrogen bonds that generate 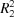
(12) ring motifs. A second inversion dimer to an adjacent mol­ecule results from C1—H1⋯O2 contacts, forming ring 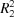
(22) **[OK?]** rings, Fig. 3 ▸.

## Database survey   

The Cambridge Structural Database (Groom & Allen, 2014 ▸) reveals only five structures of imidazole derivatives with a CH_2_COOCH_2_CH_3_ substituent on nitro­gen (Cai *et al.*, 2014 ▸; Bahnous *et al.*, 2013 ▸; Zaprutko *et al.*, 2012 ▸). Imidazoles with benzoyl substituents are slightly more common with eight occurrences (Xue *et al.*, 2014 ▸; Nagaraj *et al.*, 2012 ▸; Samanta *et al.*, 2013 ▸), while the structures of only six *p*-tolyl-substituted imidazoles are found (Bu *et al.*, 1996 ▸; Fridman *et al.*, 2006 ▸, 2009 ▸). These searches also reveal the unique nature of the mol­ecule reported here.

## Synthesis and crystallization   

The title compound was synthesized from a mixture of 2-(4-meth­oxy­phen­yl)-2-oxoacetaldehyde (1 mmol), glycine methyl ester hydro­chloride (1 mmol) and selenium dioxide (1 mmol) in a basic environment in aceto­nitrile at 373 K. Crystals suitable for X-ray investigation were obtained by solvent evaporation from the resulting solution in 33% yield.

## Refinement   

Crystal data, data collection and structure refinement details are summarized in Table 2 ▸. All H atoms were positioned geometrically and refined using a riding model, with C—H = 0.93 −0.97 Å and *U*_iso_(H) = 1.2–1.5*U*_eq_(parent C atom). The methyl group C22 of the side chain is disordered over two positions, each with a site-occupancy factor of 0.5. The atomic displacement parameters of these two C atoms are restrained to be equivalent and the C21—C22 and C21—C22′ bond distances were restrained during the refinement using DFIX commands.

## Supplementary Material

Crystal structure: contains datablock(s) I. DOI: 10.1107/S2056989016002504/sj5493sup1.cif

Structure factors: contains datablock(s) I. DOI: 10.1107/S2056989016002504/sj5493Isup2.hkl

Supporting information file. DOI: 10.1107/S2056989016002504/sj5493Isup3.cml

CCDC reference: 1452746

Additional supporting information:  crystallographic information; 3D view; checkCIF report

## Figures and Tables

**Table 1:** Hydrogen-bond geometry (Å, °)

*D*—H⋯*A*	*D*—H	H⋯*A*	*D*⋯*A*	*D*—H⋯*A*
C6—H6⋯O2	0.93	2.91	3.723 (4)	147
C1—H1*A*⋯O2^i^	0.96	2.71	3.605 (4)	155
C4—H4⋯N1^ii^	0.93	2.83	3.724 (3)	161
C19—H19*A*⋯O2^iii^	0.97	2.51	3.309 (3)	140

Symmetry codes: (i) 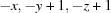; (ii) 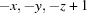; (iii) 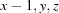.

**Table 2:** Experimental details

Crystal data	
Chemical formula	C_22_H_22_N_2_O_3_
*M*_r_	362.41
Crystal system, space group	Triclinic, *P* 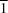
Temperature (K)	293
*a*, *b*, *c* (Å)	5.0968 (5), 13.8189 (15), 14.6993 (17)
α, β, γ (°)	71.484 (5), 84.018 (5), 82.531 (5)
*V* (Å^3^)	971.20 (18)
*Z*	2
Radiation type	Mo *K*α
μ (mm^−1^)	0.08
Crystal size (mm)	0.21 × 0.19 × 0.16

Data collection	
Diffractometer	Bruker SMART APEX CCD area-detector
No. of measured, independent and observed [*I* > 2σ(*I*)] reflections	18453, 3405, 2354
*R*_int_	0.055
(sin θ/λ)_max_ (Å^−1^)	0.595

Refinement	
*R*[*F* ^2^ > 2σ(*F* ^2^)], *wR*(*F* ^2^), *S*	0.054, 0.168, 1.07
No. of reflections	3405
No. of parameters	251
No. of restraints	2
H-atom treatment	H-atom parameters constrained
Δρ_max_, Δρ_min_ (e Å^−3^)	0.52, −0.31

Computer programs: *SMART* (Bruker, 2001 ▸), *SAINT* (Bruker, 2001 ▸), *SHELXTL/PC* (Sheldrick, 2008 ▸) and *PLATON* (Spek, 2009 ▸).

## supplementary crystallographic information

### Crystal data

**Table:**

C_22_H_22_N_2_O_3_	*Z* = 2
*M**_r_* = 362.41	*F*(000) = 384
Triclinic, *P*1	*D*_x_ = 1.239 Mg m^−^^3^
*a* = 5.0968 (5) Å	Mo *K*α radiation, λ = 0.71073 Å
*b* = 13.8189 (15) Å	Cell parameters from 2986 reflections
*c* = 14.6993 (17) Å	θ = 2.1–24.4°
α = 71.484 (5)°	µ = 0.08 mm^−^^1^
β = 84.018 (5)°	*T* = 293 K
γ = 82.531 (5)°	Block, colourless
*V* = 971.20 (18) Å^3^	0.21 × 0.19 × 0.16 mm

### Data collection

**Table:**

Bruker SMART APEX CCD area-detector diffractometer	*R*_int_ = 0.055
Radiation source: fine-focus sealed tube	θ_max_ = 25.0°, θ_min_ = 2.5°
ω scans	*h* = −6→6
18453 measured reflections	*k* = −16→16
3405 independent reflections	*l* = −17→17
2354 reflections with *I* > 2σ(*I*)	

### Refinement

**Table:**

Refinement on *F*^2^	2 restraints
Least-squares matrix: full	Hydrogen site location: inferred from neighbouring sites
*R*[*F*^2^ > 2σ(*F*^2^)] = 0.054	H-atom parameters constrained
*wR*(*F*^2^) = 0.168	*w* = 1/[σ^2^(*F*_o_^2^) + (0.0636*P*)^2^ + 0.5608*P*] where *P* = (*F*_o_^2^ + 2*F*_c_^2^)/3
*S* = 1.07	(Δ/σ)_max_ = 0.002
3405 reflections	Δρ_max_ = 0.52 e Å^−^^3^
251 parameters	Δρ_min_ = −0.30 e Å^−^^3^

### Special details

**Table:**

Geometry. All e.s.d.'s (except the e.s.d. in the dihedral angle between two l.s. planes) are estimated using the full covariance matrix. The cell e.s.d.'s are taken into account individually in the estimation of e.s.d.'s in distances, angles and torsion angles; correlations between e.s.d.'s in cell parameters are only used when they are defined by crystal symmetry. An approximate (isotropic) treatment of cell e.s.d.'s is used for estimating e.s.d.'s involving l.s. planes.

### Fractional atomic coordinates and isotropic or equivalent isotropic displacement parameters (Å^2^)

**Table:**

	*x*	*y*	*z*	*U*_iso_*/*U*_eq_	Occ. (<1)
C1	−0.6633 (7)	0.3819 (3)	0.6826 (2)	0.0754 (9)	
H1A	−0.6105	0.4476	0.6779	0.113*	
H1B	−0.6498	0.3372	0.7474	0.113*	
H1C	−0.8433	0.3901	0.6655	0.113*	
C2	−0.4852 (5)	0.3362 (2)	0.61545 (19)	0.0530 (7)	
C3	−0.5093 (5)	0.2386 (2)	0.6123 (2)	0.0568 (7)	
H3	−0.6403	0.2015	0.6517	0.068*	
C4	−0.3441 (5)	0.19537 (19)	0.55220 (19)	0.0501 (6)	
H4	−0.3636	0.1293	0.5524	0.060*	
C5	−0.1486 (5)	0.24912 (17)	0.49132 (17)	0.0426 (6)	
C6	−0.1244 (5)	0.34725 (19)	0.4938 (2)	0.0536 (7)	
H6	0.0048	0.3851	0.4539	0.064*	
C7	−0.2906 (5)	0.3891 (2)	0.5551 (2)	0.0573 (7)	
H7	−0.2705	0.4548	0.5557	0.069*	
C8	0.0360 (5)	0.19884 (17)	0.43313 (17)	0.0419 (6)	
C9	0.1875 (5)	0.10614 (18)	0.46091 (18)	0.0466 (6)	
H9	0.1835	0.0613	0.5233	0.056*	
C10	0.2885 (5)	0.16918 (17)	0.31157 (17)	0.0428 (6)	
C11	0.4033 (5)	0.1806 (2)	0.21394 (19)	0.0539 (7)	
C12	0.6016 (5)	0.0980 (2)	0.19780 (17)	0.0480 (6)	
C17	0.8022 (5)	0.0507 (2)	0.25858 (19)	0.0514 (7)	
H17	0.8186	0.0717	0.3117	0.062*	
C16	0.9772 (6)	−0.0268 (2)	0.2414 (2)	0.0614 (8)	
H16	1.1136	−0.0560	0.2821	0.074*	
C15	0.9556 (6)	−0.0622 (2)	0.1653 (2)	0.0629 (8)	
C14	0.7592 (7)	−0.0130 (3)	0.1034 (2)	0.0777 (10)	
H14	0.7426	−0.0345	0.0505	0.093*	
C13	0.5874 (6)	0.0671 (3)	0.1182 (2)	0.0709 (9)	
H13	0.4612	0.1004	0.0742	0.085*	
C18	1.1384 (8)	−0.1519 (3)	0.1513 (3)	0.0990 (13)	
H18A	1.0844	−0.1703	0.0988	0.149*	
H18B	1.1306	−0.2092	0.2089	0.149*	
H18C	1.3167	−0.1334	0.1372	0.149*	
C19	−0.0310 (5)	0.33098 (18)	0.27136 (18)	0.0488 (6)	
H19A	−0.1902	0.3538	0.3044	0.059*	
H19B	−0.0836	0.3150	0.2167	0.059*	
C20	0.1464 (5)	0.41606 (19)	0.23635 (19)	0.0510 (7)	
C21	0.2175 (8)	0.5710 (2)	0.1156 (2)	0.0923 (12)	
H21A	0.4038	0.5456	0.1198	0.111*	
H21B	0.1794	0.6196	0.1517	0.111*	
C22	0.161 (3)	0.6255 (15)	0.0101 (4)	0.119 (4)	0.5
H22A	0.2473	0.5850	−0.0289	0.179*	0.5
H22B	0.2273	0.6915	−0.0105	0.179*	0.5
H22C	−0.0267	0.6340	0.0036	0.179*	0.5
C22'	0.047 (3)	0.6433 (15)	0.0364 (5)	0.119 (4)	0.5
H22D	0.0075	0.6068	−0.0055	0.179*	0.5
H22E	0.1418	0.7005	0.0001	0.179*	0.5
H22F	−0.1156	0.6677	0.0651	0.179*	0.5
N1	0.3431 (4)	0.08765 (14)	0.38698 (14)	0.0464 (5)	
N2	0.1006 (4)	0.23871 (14)	0.33651 (14)	0.0434 (5)	
O1	0.3314 (5)	0.25504 (18)	0.14648 (15)	0.0913 (8)	
O2	0.3358 (4)	0.42182 (16)	0.27496 (17)	0.0774 (7)	
O3	0.0563 (4)	0.48554 (13)	0.15783 (13)	0.0663 (6)	

### Atomic displacement parameters (Å^2^)

**Table:**

	*U*^11^	*U*^22^	*U*^33^	*U*^12^	*U*^13^	*U*^23^
C1	0.077 (2)	0.081 (2)	0.077 (2)	0.0039 (17)	−0.0022 (17)	−0.0423 (18)
C2	0.0517 (15)	0.0546 (16)	0.0558 (16)	0.0006 (13)	−0.0118 (13)	−0.0211 (13)
C3	0.0558 (16)	0.0555 (17)	0.0601 (17)	−0.0134 (13)	0.0026 (13)	−0.0185 (14)
C4	0.0553 (15)	0.0386 (13)	0.0585 (16)	−0.0095 (12)	−0.0050 (13)	−0.0155 (12)
C5	0.0453 (13)	0.0361 (12)	0.0458 (14)	−0.0031 (10)	−0.0120 (11)	−0.0094 (11)
C6	0.0531 (15)	0.0392 (14)	0.0681 (18)	−0.0111 (12)	−0.0003 (13)	−0.0149 (13)
C7	0.0617 (17)	0.0408 (14)	0.0759 (19)	−0.0036 (13)	−0.0105 (15)	−0.0259 (14)
C8	0.0475 (14)	0.0331 (12)	0.0450 (14)	−0.0075 (10)	−0.0073 (11)	−0.0092 (11)
C9	0.0595 (16)	0.0362 (13)	0.0405 (14)	−0.0052 (11)	−0.0059 (12)	−0.0060 (11)
C10	0.0459 (14)	0.0346 (12)	0.0447 (14)	−0.0027 (10)	−0.0072 (11)	−0.0071 (11)
C11	0.0557 (16)	0.0496 (15)	0.0467 (15)	−0.0009 (12)	−0.0049 (12)	−0.0025 (12)
C12	0.0499 (15)	0.0515 (15)	0.0401 (14)	−0.0078 (12)	−0.0016 (11)	−0.0099 (11)
C17	0.0535 (15)	0.0524 (15)	0.0478 (15)	−0.0058 (13)	−0.0060 (12)	−0.0136 (12)
C16	0.0600 (17)	0.0582 (17)	0.0561 (17)	0.0031 (14)	−0.0026 (13)	−0.0077 (14)
C15	0.0662 (19)	0.0526 (17)	0.0669 (19)	−0.0124 (14)	0.0146 (15)	−0.0175 (15)
C14	0.074 (2)	0.109 (3)	0.070 (2)	−0.016 (2)	0.0051 (17)	−0.055 (2)
C13	0.0578 (18)	0.106 (3)	0.0529 (17)	0.0029 (17)	−0.0105 (14)	−0.0325 (17)
C18	0.116 (3)	0.063 (2)	0.112 (3)	−0.003 (2)	0.032 (2)	−0.032 (2)
C19	0.0471 (14)	0.0397 (13)	0.0500 (15)	0.0019 (11)	−0.0091 (11)	−0.0010 (11)
C20	0.0540 (16)	0.0381 (14)	0.0522 (15)	0.0030 (12)	−0.0057 (13)	−0.0041 (12)
C21	0.133 (3)	0.0462 (18)	0.085 (2)	−0.0256 (19)	−0.012 (2)	0.0059 (17)
C22	0.199 (13)	0.099 (7)	0.050 (4)	−0.063 (7)	0.004 (6)	0.004 (6)
C22'	0.199 (13)	0.099 (7)	0.050 (4)	−0.063 (7)	0.004 (6)	0.004 (6)
N1	0.0566 (13)	0.0345 (11)	0.0443 (12)	−0.0018 (9)	−0.0086 (10)	−0.0061 (9)
N2	0.0462 (11)	0.0331 (10)	0.0448 (12)	−0.0018 (9)	−0.0081 (9)	−0.0029 (9)
O1	0.1031 (18)	0.0828 (16)	0.0517 (12)	0.0282 (14)	0.0041 (12)	0.0128 (11)
O2	0.0675 (13)	0.0612 (13)	0.0934 (16)	−0.0175 (11)	−0.0261 (12)	0.0010 (11)
O3	0.0886 (14)	0.0412 (10)	0.0577 (12)	−0.0074 (10)	−0.0135 (10)	0.0037 (9)

### Geometric parameters (Å, º)

**Table:**

C1—C2	1.503 (4)	C16—C15	1.375 (4)
C1—H1A	0.9600	C16—H16	0.9300
C1—H1B	0.9600	C15—C14	1.383 (4)
C1—H1C	0.9600	C15—C18	1.506 (4)
C2—C7	1.377 (4)	C14—C13	1.379 (4)
C2—C3	1.386 (4)	C14—H14	0.9300
C3—C4	1.377 (4)	C13—H13	0.9300
C3—H3	0.9300	C18—H18A	0.9600
C4—C5	1.389 (3)	C18—H18B	0.9600
C4—H4	0.9300	C18—H18C	0.9600
C5—C6	1.390 (3)	C19—N2	1.459 (3)
C5—C8	1.463 (3)	C19—C20	1.503 (4)
C6—C7	1.381 (4)	C19—H19A	0.9700
C6—H6	0.9300	C19—H19B	0.9700
C7—H7	0.9300	C20—O2	1.193 (3)
C8—C9	1.371 (3)	C20—O3	1.325 (3)
C8—N2	1.372 (3)	C21—O3	1.462 (4)
C9—N1	1.349 (3)	C21—C22	1.534 (2)
C9—H9	0.9300	C21—C22'	1.535 (2)
C10—N1	1.329 (3)	C21—H21A	0.9700
C10—N2	1.373 (3)	C21—H21B	0.9700
C10—C11	1.460 (4)	C22—H22A	0.9600
C11—O1	1.227 (3)	C22—H22B	0.9600
C11—C12	1.484 (4)	C22—H22C	0.9600
C12—C13	1.379 (4)	C22'—H22D	0.9600
C12—C17	1.384 (3)	C22'—H22E	0.9600
C17—C16	1.373 (4)	C22'—H22F	0.9600
C17—H17	0.9300		
			
C2—C1—H1A	109.5	C14—C15—C18	121.8 (3)
C2—C1—H1B	109.5	C13—C14—C15	121.5 (3)
H1A—C1—H1B	109.5	C13—C14—H14	119.2
C2—C1—H1C	109.5	C15—C14—H14	119.2
H1A—C1—H1C	109.5	C12—C13—C14	120.4 (3)
H1B—C1—H1C	109.5	C12—C13—H13	119.8
C7—C2—C3	117.3 (3)	C14—C13—H13	119.8
C7—C2—C1	121.5 (3)	C15—C18—H18A	109.5
C3—C2—C1	121.2 (3)	C15—C18—H18B	109.5
C4—C3—C2	121.6 (3)	H18A—C18—H18B	109.5
C4—C3—H3	119.2	C15—C18—H18C	109.5
C2—C3—H3	119.2	H18A—C18—H18C	109.5
C3—C4—C5	120.8 (2)	H18B—C18—H18C	109.5
C3—C4—H4	119.6	N2—C19—C20	111.7 (2)
C5—C4—H4	119.6	N2—C19—H19A	109.3
C4—C5—C6	117.8 (2)	C20—C19—H19A	109.3
C4—C5—C8	119.7 (2)	N2—C19—H19B	109.3
C6—C5—C8	122.3 (2)	C20—C19—H19B	109.3
C7—C6—C5	120.5 (2)	H19A—C19—H19B	107.9
C7—C6—H6	119.7	O2—C20—O3	124.9 (2)
C5—C6—H6	119.7	O2—C20—C19	125.1 (2)
C2—C7—C6	121.9 (2)	O3—C20—C19	109.9 (2)
C2—C7—H7	119.1	O3—C21—C22	111.1 (9)
C6—C7—H7	119.1	O3—C21—C22'	102.4 (9)
C9—C8—N2	104.8 (2)	O3—C21—H21A	109.4
C9—C8—C5	129.3 (2)	C22—C21—H21A	109.4
N2—C8—C5	125.8 (2)	O3—C21—H21B	109.4
N1—C9—C8	112.0 (2)	C22—C21—H21B	109.4
N1—C9—H9	124.0	H21A—C21—H21B	108.0
C8—C9—H9	124.0	C21—C22—H22A	109.5
N1—C10—N2	111.2 (2)	C21—C22—H22B	109.5
N1—C10—C11	124.2 (2)	H22A—C22—H22B	109.5
N2—C10—C11	124.5 (2)	C21—C22—H22C	109.5
O1—C11—C10	120.8 (2)	H22A—C22—H22C	109.5
O1—C11—C12	120.8 (2)	H22B—C22—H22C	109.5
C10—C11—C12	118.3 (2)	C21—C22'—H22D	109.5
C13—C12—C17	118.2 (3)	C21—C22'—H22E	109.5
C13—C12—C11	118.7 (2)	H22D—C22'—H22E	109.5
C17—C12—C11	123.2 (2)	C21—C22'—H22F	109.5
C16—C17—C12	120.8 (3)	H22D—C22'—H22F	109.5
C16—C17—H17	119.6	H22E—C22'—H22F	109.5
C12—C17—H17	119.6	C10—N1—C9	105.0 (2)
C17—C16—C15	121.5 (3)	C8—N2—C10	106.94 (18)
C17—C16—H16	119.2	C8—N2—C19	125.8 (2)
C15—C16—H16	119.2	C10—N2—C19	126.8 (2)
C16—C15—C14	117.4 (3)	C20—O3—C21	114.8 (2)
C16—C15—C18	120.8 (3)		
			
C7—C2—C3—C4	0.7 (4)	C17—C16—C15—C14	3.4 (4)
C1—C2—C3—C4	−178.8 (3)	C17—C16—C15—C18	−176.0 (3)
C2—C3—C4—C5	−0.9 (4)	C16—C15—C14—C13	−1.4 (5)
C3—C4—C5—C6	0.6 (4)	C18—C15—C14—C13	178.0 (3)
C3—C4—C5—C8	175.8 (2)	C17—C12—C13—C14	3.8 (4)
C4—C5—C6—C7	0.0 (4)	C11—C12—C13—C14	−176.6 (3)
C8—C5—C6—C7	−175.1 (2)	C15—C14—C13—C12	−2.3 (5)
C3—C2—C7—C6	−0.2 (4)	N2—C19—C20—O2	−20.6 (4)
C1—C2—C7—C6	179.4 (3)	N2—C19—C20—O3	161.4 (2)
C5—C6—C7—C2	−0.2 (4)	N2—C10—N1—C9	−0.2 (3)
C4—C5—C8—C9	−51.0 (4)	C11—C10—N1—C9	−177.1 (2)
C6—C5—C8—C9	124.0 (3)	C8—C9—N1—C10	−0.3 (3)
C4—C5—C8—N2	131.5 (3)	C9—C8—N2—C10	−0.7 (3)
C6—C5—C8—N2	−53.5 (4)	C5—C8—N2—C10	177.3 (2)
N2—C8—C9—N1	0.7 (3)	C9—C8—N2—C19	172.2 (2)
C5—C8—C9—N1	−177.2 (2)	C5—C8—N2—C19	−9.8 (4)
N1—C10—C11—O1	175.3 (3)	N1—C10—N2—C8	0.6 (3)
N2—C10—C11—O1	−1.2 (4)	C11—C10—N2—C8	177.5 (2)
N1—C10—C11—C12	−2.5 (4)	N1—C10—N2—C19	−172.3 (2)
N2—C10—C11—C12	−179.0 (2)	C11—C10—N2—C19	4.7 (4)
O1—C11—C12—C13	−39.9 (4)	C20—C19—N2—C8	111.7 (3)
C10—C11—C12—C13	137.9 (3)	C20—C19—N2—C10	−76.8 (3)
O1—C11—C12—C17	139.7 (3)	O2—C20—O3—C21	3.5 (4)
C10—C11—C12—C17	−42.6 (4)	C19—C20—O3—C21	−178.5 (2)
C13—C12—C17—C16	−1.8 (4)	C22—C21—O3—C20	160.0 (6)
C11—C12—C17—C16	178.6 (2)	C22'—C21—O3—C20	−172.9 (6)
C12—C17—C16—C15	−1.9 (4)		

### Hydrogen-bond geometry (Å, º)

**Table:**

*D*—H···*A*	*D*—H	H···*A*	*D*···*A*	*D*—H···*A*
C6—H6···O2	0.93	2.91	3.723 (4)	147
C1—H1*A*···O2^i^	0.96	2.71	3.605 (4)	155
C4—H4···N1^ii^	0.93	2.83	3.724 (3)	161
C19—H19*A*···O2^iii^	0.97	2.51	3.309 (3)	140

Symmetry codes: (i) −*x*, −*y*+1, −*z*+1; (ii) −*x*, −*y*, −*z*+1; (iii) *x*−1, *y*, *z*.
